# DNA and RNA metabarcoding reveal shared dominant seed-borne fungi

**DOI:** 10.1186/s40793-026-00923-6

**Published:** 2026-07-03

**Authors:** Iva Franić, Patrick Sherwood, Kinga Stolarek, René Eschen, Jana Orbach, Simone Prospero, Michelle Cleary

**Affiliations:** 1 Swiss Federal Institute for Forest, Snow and Landscape Research WSL, Birmensdorf, Switzerland; 2 Swedish University of Agricultural Sciences, Southern Swedish Forest Research Centre, Alnarp, Sweden; 3 CABI, Delémont, Switzerland; 4 Federal Office for Agriculture, Bern, Switzerland

**Keywords:** Microbiome, High-throughput sequencing, Viable community, Metabolic activity, Relic DNA, Phytosanitary risk

## Abstract

**Background:**

Tree seeds harbor diverse fungal communities, including both pathogens and mutualists, that can influence plant health. These communities comprise living, metabolically active organisms as well as dormant or dead cells. Because only active fungi interact with their hosts, distinguishing active from inactive taxa is crucial, especially for environmental and phytosanitary monitoring. Traditional culturing methods capture living fungi but account for only a small fraction of the total fungal diversity. Currently, these methods are increasingly replaced by high-throughput DNA metabarcoding, which detects a broader range of taxa. However, DNA persists after cell death and occurs in dormant cells, preventing distinction between active and inactive fungi. In contrast, RNA metabarcoding may better reflect living fungal communities than the other two methods, though its use in assessing plant-associated fungi remains underexplored. We used culturing, DNA-, and RNA-based metabarcoding to compare fungal communities associated with seeds of three key European tree species (*Fagus sylvatica*, *Abies alba*, *Pinus sylvestris*).

**Results:**

Dominant fungal communities in seeds were strongly shaped by host species identity and were largely shared across DNA and RNA metabarcoding datasets, with roughly half of the most abundant genera detected by both methods. Differences between DNA- and RNA-derived communities were predominantly associated with rare taxa in the RNA dataset, although distinguishing true biological signals from noise introduced by different methodological workflows remains challenging. Several cultured genera, likely both abundant and metabolically active, were consistently detected by both approaches.

**Conclusions:**

These results highlight the complementary nature of the three methods for characterising seed-associated fungi. Combining culturing, DNA- and RNA-based metabarcoding may provide the most comprehensive assessment of fungal diversity, while RNA metabarcoding alone offers a promising opportunity to identify the active members of fungal communities for improved environmental and phytosanitary monitoring.

**Supplementary Information:**

The online version contains supplementary material available at https://doi.org/10.1186/s40793-026-00923-6.

## Background

Seeds are essential for plant reproduction and dispersal and act as selective microbial habitats. Packed with oils, starches, and sugars, their resources are protected by seed coats and chemical defences [1], allowing access only to specialized microorganisms [2]. Molecular studies show that tree seeds host diverse fungi [3], including plant pathogens [4–6], and fungi that protect plants against biotic and abiotic stresses [7]. However, few of these fungi may be metabolically active, while the remainder are dead or dormant. Differentiating these fractions is key to understanding the roles of seed-associated fungi in their hosts and their impact on tree health.

Plant-associated fungi have been traditionally studied by growing them from plant tissues on nutrient media and identifying the isolates based on morphology and/or with molecular techniques [8]. This approach has been widely used to explore fungal biodiversity and ecology [9–11] and remains essential for obtaining living cultures, describing new species and conducting phenotyping assays (e.g., host range, virulence, optimal growth temperatures). For studying new host–pathogen associations, living cultures are also necessary to fulfil Koch’s postulates. However, culturing captures only a subset of fungal diversity, mainly abundant fungi, excluding taxa that are difficult or impossible to isolate [3], but which may affect tree health [12]. Moreover, culturing is time-consuming and costly [13], limiting its usefulness for large-scale applications such as environmental monitoring or phytosanitary screening.

Recent advances in high-throughput sequencing (HTS) have enabled the rapid and sensitive analysis of many samples simultaneously. Amplicon-based HTS (i.e., metabarcoding) has become widely used for profiling fungal communities from various substrates, including plant tissues, to address questions related to their diversity and biosecurity [14, 15]. This approach typically targets the internal transcribed spacer (ITS) region of the nuclear ribosomal RNA (rRNA) gene cluster, a universal fungal barcode [16]. Sequencing platforms such as Illumina or PacBio are then used to characterise fungi from DNA extracted directly from bulk, host or environmental samples. However, DNA metabarcoding cannot distinguish living, metabolically active from dead or dormant organisms [14]. For example, Carini et al. [17] showed that over 40% of ITS sequences in soil belong to dead organisms, inflating diversity and skewing community profiles. This raises concerns when using DNA metabarcoding to study fungal responses to environmental change or in biosecurity [17, 18], where only viable organisms pose a biosecurity threat that requires mitigation measures. Additionally, distinguishing dead from living organisms is essential for evaluating the effectiveness of phytosanitary treatments. Unlike DNA, RNA generally has a shorter persistence after cell death, although its stability varies among RNA types. Messenger RNA is present only in metabolically active cells and degrades rapidly after cell death [19, 20], thus reflecting the actively growing community. Mature rRNA can persist in inactive or dormant cells for a limited time, capturing both active and inactive populations [21, 22]. In fungi, the nuclear rRNA gene cluster is transcribed into a precursor rRNA that includes the ITS region. Targeting this precursor transcript preferentially detects cells that are actively transcribing rRNA genes during ribosome synthesis [23, 24], potentially targeting living, active community. While the potential of using RNA for improved biological monitoring has been recognised in environmental fields such as water research [25], the approach remains largely underexplored, including for detecting active fungi in plant samples.

Studies using DNA and RNA metabarcoding revealed different total and active microbial communities in temperate soil [26], wood [27], ballast water [18, 28] and ocean [29]. However, other studies found no differences. For example, 92% of fungal taxa in *Picea abies* needles overlapped between DNA and RNA datasets [30], and similar patterns were observed in Arctic soil [31]. The differences between total and active fungal communities may depend on environmental factors such as resource availability and temperature, which influence fungal metabolism [32, 33] and DNA/RNA turnover [34]. Methodological biases may also contribute to the observed discrepancies, highlighting the need to assess how well these two approaches reflect fungal assemblages, especially when distinguishing active fungi from dead or dormant ones is critical for ecological inference and biosecurity.

In this study, we characterised fungal communities associated with commercially traded seeds of three ecologically and economically important European tree species—common beech (*Fagus sylvatica* L.), silver fir (*Abies alba* Mill.), and Scots pine (*Pinus sylvestris* L.)—using traditional culturing and DNA- and RNA-based metabarcoding. We assessed incidence-based and abundance-weighted alpha diversity (i.e., OTU richness and Inverse Simpson index, respectively) and beta diversity (i.e., Sørensen and Morisita–Horn dissimilarities, respectively) across tree species and molecular methods. We also compared taxonomic and lifestyle composition of fungal communities assessed with DNA and RNA metabarcoding within each tree species and compared fungal communities detected by each metabarcoding method with those from culturing. We hypothesised that DNA metabarcoding would reveal greater diversity than RNA metabarcoding, as RNA-based communities represent the active subset of the DNA-detected communities. Moreover, we hypothesised that fungi obtained by culturing would more closely resemble RNA-based communities than DNA-based ones, because culturing primarily recovers living, metabolically active fungi at the time of isolation.

## Methods

### Study material

A total of 16 seed lots (i.e., batches of seeds from specific locations and tree species; *F. sylvatica* (n = 6), *A. alba* (n = 5), *P. sylvestris* (n = 5)) were obtained in winter 2019 from European commercial seed suppliers. Seeds were stored at 4 °C until fungal assessment using culturing, DNA- and RNA-metabarcoding in spring 2020.

### Fungal assessment by culturing

Seeds were surface sterilized by subsequent immersion in 70% ethanol (1 min), 1% sodium hypochlorite (5 min), 70% ethanol (30 s), and sterile water (30 s) [35], then dried in a laminar flow hood. For fungal assessment, 90 seeds per seed lot were crushed with a sterile pestle and placed on nutrient media containing streptomycin (0.05 mg/mL). To capture a broad range of fungi, three media with varying nutrient content were used—water agar (WA 15 g/L; VWR Chemicals, Solon, Ohio, USA), malt extract agar (MEA; ME 30 g/L; Duchefa Biochemie, Haarlem, the Netherlands & WA 15 g/L; VWR Chemicals) and potato dextrose agar (PDA 39 g/L; Merck KGaA, Darmstadt, Germany). Thirty seeds were plated per tree species x medium combination. Plates were checked every five days for 30 days. Emerging colonies were transferred to PDA (39 g/L; Merck KGaA) to obtain pure cultures. Isolates were grouped based on macromorphological traits (e.g., colour, texture, form, margin).

One isolate per morphotype was selected for DNA extraction. Mycelia were scraped from agar, freeze-dried overnight, and DNA was extracted using the E.Z.N.A.® SP Plant DNA Kit (Omega Bio-Tek, Norcross, Georgia, USA) following the manufacturer’s instructions. DNA concentrations were measured using a DS-11 UV–Vis Spectrophotometer (DeNovix, Wilmington, Delaware, USA) and diluted to 5 ng/µl. DNA extracts with concentrations below 5 ng/µl were not diluted. The ITS region was amplified in 25 µl reactions, containing 2 µl DNA, 8.5 µl nuclease-free H_2_O, 12.5 µl DreamTaqPCR Master Mix (2X), and 1 µl each of ITS1 (F: TCCGTAGGTGAACCTGCGG) and ITS4 (R: TCCTCCGCTTATTGATATGC) primers [36]. PCRs were run on an Eppendorf Mastercycler (Eppendorf, Hamburg, Germany) under the following conditions: 95 °C for 2 min; 35 cycles of 95 °C for 1 min; 55 °C for 45 s; 72 °C for 1.5 min; final extension at 72 °C for 5 min. Amplification success was confirmed by gel electrophoresis. PCR products were purified and Sanger-sequenced by Macrogen-Europe (Amsterdam, the Netherlands) using the same primers as in PCRs.

Forward and reverse sequences were trimmed and assembled into consensus sequences using CLC Main Workbench 26 (Qiagen, Aarhus, Denmark). Study sequences were compared against the National Centre for Biotechnology Information (NCBI) core nucleotide (nt) database using MEGABLAST [37, 38], excluding sequences annotated as uncultured or originating from environmental samples, to ensure reliable taxonomic assignment.

### Fungal assessment by metabarcoding

Fungal communities were analysed from 16 seed lots, each consisting of 30 pooled seeds, using DNA and RNA metabarcoding. Seeds were surface sterilised as for culturing, then ground in liquid nitrogen using mortars and pestles treated with RNase *AWAY*™ Surface Decontaminant (Thermo Fisher Scientific, Waltham, Massachusetts, USA). DNA was extracted using the E.Z.N.A.® SP Plant DNA Kit (Omega Bio-Tek) following the manufacturer’s instructions. DNA quantity was determined with a Qubit dsDNA BR Assay kit on a Qubit 3.0 Fluorometer (Thermo Fisher Scientific). Extraction blanks were not included in the DNA extraction workflow which limits the ability to assess potential background contamination and should be considered when interpreting low-abundance taxa. The ITS2 region was amplified using primers ITS3 (F: GCATCGATGAAGAACGCAGC) and ITS4 (R: TCCTCCGCTTATTGATATGC) [36]. All PCR reactions were carried out with 15 µL of Phusion High—Fidelity PCR Master Mix (Thermo Fisher Scientific), 0.2 µM of forward and reverse primers, and 10 ng template DNA. Thermal cycling consisted of initial denaturation at 98 ℃ for 1 min, 30 cycles of denaturation at 98 ℃ for 10 s, annealing at 50 ℃ for 30 s, and elongation at 72 ℃ for 30 s and 72 ℃ for 5 min. All PCR reactions included a positive control with a known DNA template and a negative control lacking template DNA to confirm reaction success and monitor for potential contamination. Libraries were prepared and sequenced on an Illumina MiSeq (2 × 250 bp) at Novogene (Cambridge, UK).

RNA was extracted using the E.Z.N.A.® Plant RNA kit (Omega Bio-Tek) following the manufacturer`s protocol for difficult samples, from the same ground seed pool used for DNA extractions. Multiple extractions per sample were pooled to obtain sufficient RNA for complementary DNA (i.e., cDNA) synthesis. As for the DNA extraction workflow, extraction blanks were not included for RNA, resulting in the same limitation as described above. After pooling, RNA quantity was measured using the Qubit™ RNA Broad Range (BR) Assay Kit with Qubit 3.0 Fluorometer (Thermo Fisher Scientific), and quality was assessed using the Agilent RNA 6000 Pico Kit and Agilent 2100 Bioanalyzer (Agilent, Santa Clara, California, USA). DNA contamination was removed using TURBO DNase (Invitrogen™, Thermo Fisher Scientific) with a 60 min incubation (4 U/reaction), confirmed by 35-cycle ITS PCR (ITS1/ITS4 primers; [36]) as described above and the absence of bands in 1.2% TAE agarose gel electrophoresis. RNA was cleaned and concentrated with the RNeasy MinElute Cleanup Kit (QIAGEN, Hilden, Germany), and cDNA synthesised using the SuperScript™ IV First-Strand System (Invitrogen™, Thermo Fisher Scientific) with random hexamer primers and RNase H treatment. The resulting cDNA was purified with AMPure XP beads (1:4 bead:sample ratio) and stored at − 80°. The ITS2 region was amplified using ITS3 and ITS4 primers [36] as described for DNA. Library preparation and Illumina NovaSeq 6000 sequencing (2 × 250 bp) were done at BMKGENE (Münster, Germany).

Samples were demultiplexed by the sequencing facility, yielding separate forward and reverse FASTQ files for each sample and dataset (i.e., DNA and RNA). Bioinformatic processing was performed using a custom QIIME2-based pipeline [39]. Adapters and primers were removed with Cutadapt (i.e., error rate 0.1; [40]). Quality filtering, denoising, read merging and chimera removal were conducted using DADA2 [41] with default parameters. Amplicon sequence variants of both DNA and RNA datasets were clustered into OTUs at 97% similarity using VSEARCH [42], and taxonomic assignment of the merged dataset was performed with a Naïve Bayes classifier [43] in QIIME2 against the UNITE v8.2 database [44]. Taxonomic assignments at species level were not considered, because species identification based on the ITS2 region is often unreliable, particularly for certain fungal groups [45].

### Fungal lifestyle assignments

Fungal lifestyles were assigned to OTUs based on genus-level matches using the *FungalTraits* database [46], referencing the “primary_lifestyle” column. The “plant pathogen” category was retained as is, while the following classification adjustments were made:

1. “ectomycorrhizal”, “arbuscular_mycorrhizal” = “mycorrhiza”,
2. “foliar_endophyte”, “root_endophyte” = “endophyte”,
3. “soil_saprotroph”, “dung_saprotroph”, “litter_saprotroph”, “nectar/tap_saprotroph”, “wood_saprotroph”, “pollen_saprotroph”, “unspecified_saprotroph” = ”saprotroph”,
4. “mycoparasite”, “animal_parasite”, “epiphyte”, “lichen_parasite”, “algal_parasite”, “sooty_mold”, “lichenized” = “other”.

### Statistical analyses

All analyses were performed using R Statistical software [47].

To account for differences in sequencing depth (i.e., number of reads per sample ranged from 7,939 to 1,179,119 with a mean ± se of 48,663 ± 6,590) and retain all the samples, the dataset was rarefied to a minimum number of reads per sample (i.e., 7,939 reads in a DNA sample of *P. sylvestris*; *Rarefy* function, GUniFrac package; [48]) prior to alpha and beta diversity analyses [49]. Alpha and beta diversity analyses were conducted using incidence-based and abundance-weighted metrices. While incidence-based diversity measures capture overall diversity patterns by giving equal weight to all taxa regardless of abundance, abundance-weighted measures emphasize patterns driven by dominant taxa by assigning greater weight to abundant taxa.

Alpha diversity was assessed using OTU richness (i.e., incidence-based metric) and Inverse Simpson Index (i.e., abundance-weighted metric; [50]), calculated using the *hill_taxa* function from the package hillR [51, 52]. Alpha diversity metrics (i.e., response variables) were compared between explanatory variables—the methods (i.e., DNA and RNA metabarcoding) and tree species (i.e., *F. sylvatica*, *A. alba* and *P. sylvestris*). To account for overdispersion, OTU richness was analysed using a negative binomial generalized linear model (i.e., *glm.nb* function, MASS package; [53]). Inverse Simpson Index was modelled with a gamma distribution generalized linear model using log link (i.e., *glm* function, stats package; [47]) due to its non-integer nature. Models with and without the interaction term were compared based on Akaike information criterion (AIC). Models without the interaction term were retained (i.e., lower AIC) and further evaluated for factor significance (i.e., *Anova* function, car package; [54]). Estimated marginal means for significant factors and pairwise comparisons were computed with the function *emmeans* from the emmeans package [55].

Beta diversity was assessed using Sørensen dissimilarity (i.e., incidence-based metric), and Morisita–Horn dissimilarity (i.e., abundance-weighted metric). Pairwise distances between samples were calculated using the *vegdist* function from the vegan package [56]. Overall effects of method and tree species (i.e., explanatory variables) on fungal community composition (i.e., Sørensen and Morisita–Horn dissimilarity; response variables) were tested using Permutational multivariate ANOVA with 999 permutations (i.e., PERMANOVA; adonis function, vegan package; [56]), without interaction term to match alpha diversity models. Differences in fungal community composition between the methods and tree species were visualized using non-metric multidimensional scaling (i.e., NMDS; *metaMDS* function, vegan package; [56]).

Homogeneity of group dispersions was assessed using the *betadisper* function from the vegan package [56] for both Sørensen and Morisita–Horn dissimilarity indices, and for the two factors (i.e., method and tree species**)**. Differences in dispersion were tested for significance using the *permutest* function with 999 permutations (vegan package; [56]).

As PERMANOVA revealed significant differences in fungal communities among tree species (see Results), taxonomic composition was assessed separately for each tree species and method, using a non-rarefied dataset to preserve full abundance information across samples [57]. We focused on reads assigned to fungal genera, enabling inference of ecological roles and host associations. First, the number of reads associated with each distinct fungal genus was calculated for every method and tree species. The genera were then ranked by read count, and the ten most abundant genera per method and tree species were selected. All remaining reads were grouped as either “Other” (i.e., reads assigned to genera outside the top ten) or “Unassigned” (i.e., reads not assigned to any genus). Relative abundances of the top ten genera, and genera classified as “Other” or “Unassigned” were plotted across the methods and tree species. In addition, indicator species analysis was performed using the *multipatt* function with default parameters (indicspecies package; [58]) to identify genera strongly associated with either the DNA or RNA dataset for each tree species, using all genera in the non-rarefied dataset. Statistical significance was assessed using 9,999 permutations. False discovery rate–adjusted p values were calculated using the *p.adjust* function (stats package; [47]). Due to the small sample size and exploratory nature of the analysis, taxa with unadjusted p values < 0.05 were considered statistically significant, as most associations were no longer significant after adjustment (Supplementary Table 1).

Variation in fungal lifestyles was evaluated using all non-rarefied reads assigned to a specific lifestyle category or classified as “Unassigned”. For each method and tree species, the relative abundance of reads associated with each lifestyle were calculated and plotted.

Finally, we identified fungal genera shared between culturing and each metabarcoding dataset, as well as those unique to each dataset. This was done for all tree species together to assess the overall trend, and for each tree species separately, to assess the differences across tree species.

## Results

Of the 3,167,108 non-singleton reads assigned to 1,797 OTUs in the merged DNA and RNA dataset, roughly 50% were fungal (1,557,227 reads and 942 OTUs). Rarefaction curves approached saturation, indicating that sequencing depth captured most fungal OTUs across samples (Supplementary Fig. 1). As shown in Table 1, over 60% of fungal reads originated from *A. alba*, with the remainder being similarly distributed between the other two species. Overall read counts were similar between DNA and RNA datasets, except in *P. sylvestris*, where DNA reads were nearly twice as high as in the other species. *Pinus sylvestris* also contained twice as many OTUs as *A. alba*, with *F. sylvatica* falling between the two. Across tree species, RNA datasets consistently contained three- to fourfold more OTUs than DNA datasets. The rarefied dataset, consisting of 254,048 non-singleton reads (7,939 per sample) assigned to 872 OTUs, showed a similar pattern, with the RNA dataset yielding two- to threefold more OTUs than the DNA dataset.

**Table 1:** Number of fungal reads and OTUs across three tree species and DNA and RNA metabarcoding datasets

Tree species	Reads			OTUs		
	DNA	RNA	All data	DNA	RNA	All data
*F. sylvatica* (n = 6)	105,396	177,420	282,816	108	361	417
	(47,634)	(47,634)	(95,268)	(102)	(342)	(392)
*A. alba* (n = 5)	444,834	522,881	967,715	93	232	277
	(39,695)	(39,695)	(79,390)	(72)	(173)	(202)
*P. sylvestris* (n = 5)	212,820	93,876	306,696	152	519	628
	(39,695)	(39,695)	(79,390)	(139)	(512)	(610)
Total	763,050	794,177	1,557,227	247	801	942
	(127,024)	(127,024)	(254,048)	(215)	(512)	(872)

Number of fungal reads and OTUs in samples belonging to the three tree species (i.e., *F. sylvatica*, *A. alba*, *P. sylvestris*; numbers of seed lots are indicated in brackets) are indicated. Numbers are shown for the DNA and RNA datasets and for the combined dataset (i.e., all data) considering all fungal reads and rarefied fungal reads (in the brackets)

### Alpha diversity

**Fig. 1:**
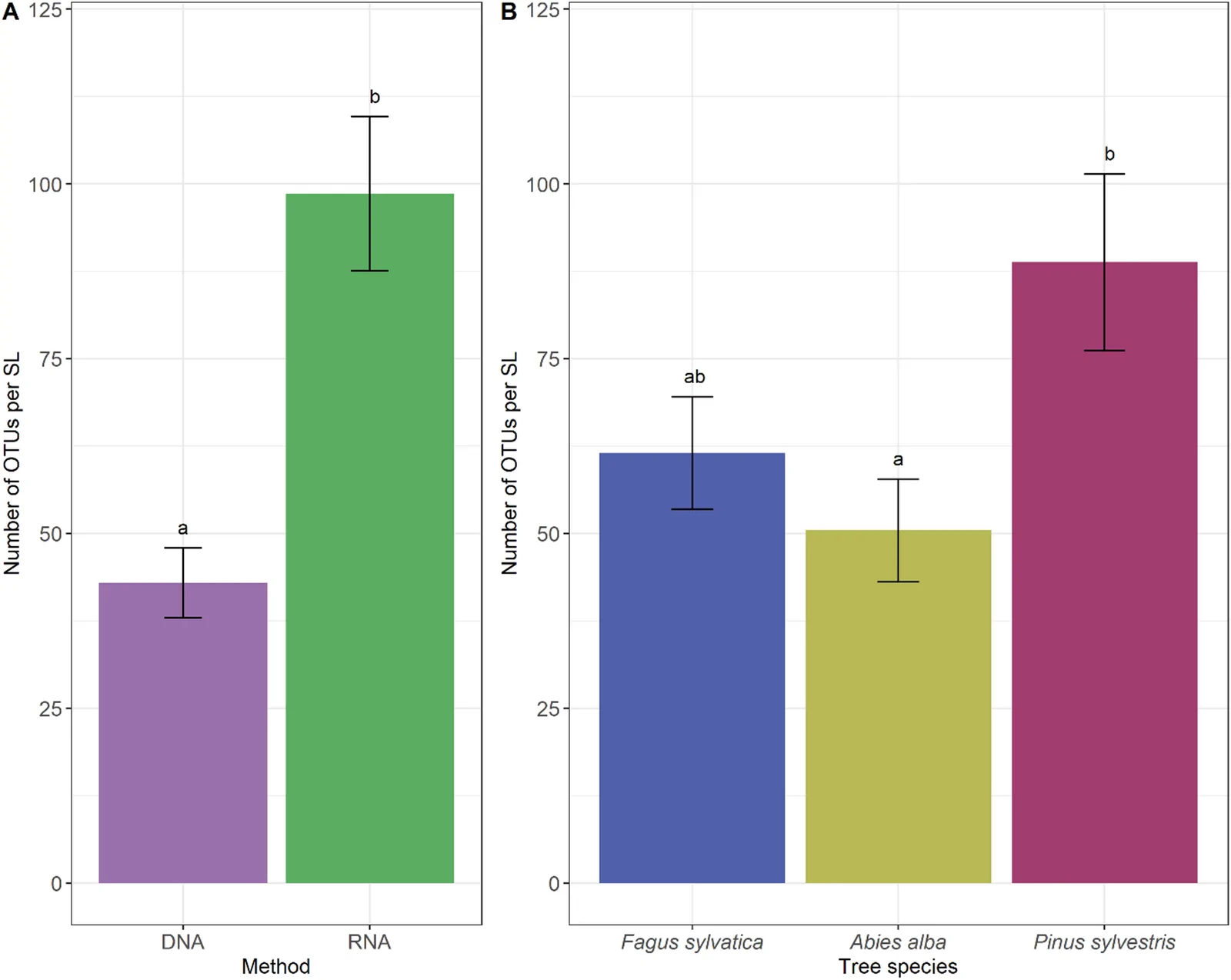
OTU richness per seed lot across metabarcoding methods and tree species. OTU richness (i.e., number of observed OTUs) per seed lot comparing different methods (**A**) and tree species (**B**) is shown. Bars represent estimated means ± standard errors (SE). The model was run on the rarefied dataset. Different letters above the bars indicate statistically significant differences (*p* < 0.05)

RNA metabarcoding revealed approximately twice as many OTUs per sample compared to DNA metabarcoding (Fig. 1A; χ2 = 25.95, df = 1, *p* < 0.001). This pattern persisted for abundance-weighted alpha diversity (i.e., Inverse Simpson Index; χ2 = 17.90, df = 1, *p* < 0.001), although the mean values of the Inverse Simpson Index were roughly ten times lower than those of OTU richness (Supplementary Fig. 2A).

Overall, OTU richness per sample differed significantly between tree species (Fig. 1B; χ2 = 8.18, df = 1, *p* < 0.05), with the lowest richness observed in *A. alba* samples and the highest in *P. sylvestris* samples. A similar pattern was observed for the abundance-weighted diversity (χ2 = 15.12, df = 2, *p* < 0.001) which was around ten times lower than OTU richness—*F. sylvatica* and *P. sylvestris* exhibited more than twice the diversity of *A. alba* (Supplementary Fig. 2B).

### Beta diversity

**Fig. 2:**
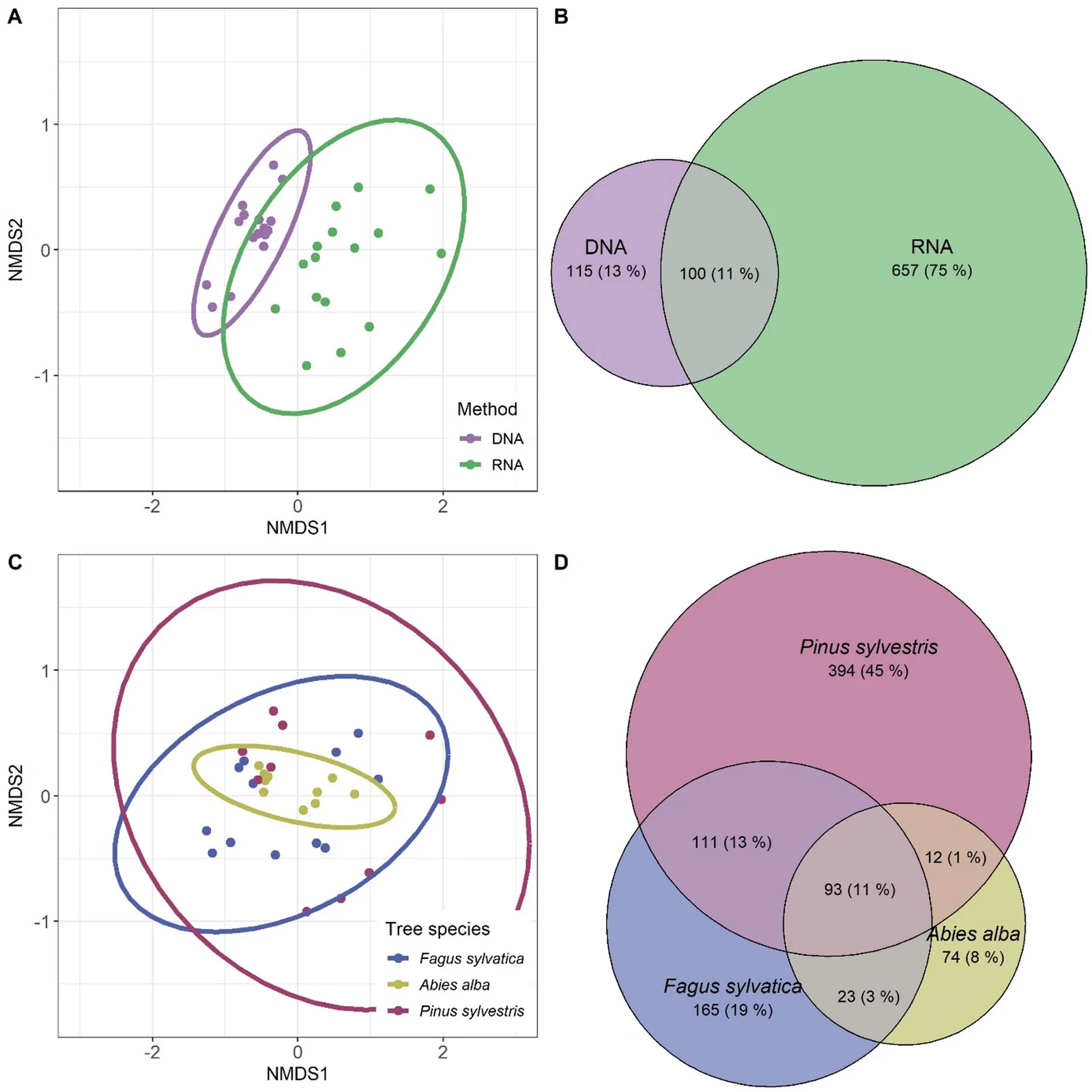
Differences in fungal community composition across metabarcoding methods and tree species. Differences in fungal community composition between samples assessed with DNA and RNA metabarcoding (**A**, **B**) and samples belonging to different tree species (**C**, **D**) are shown. Non-metric multidimensional scaling ordination plots (**A**, **C**) are based on Sørensen distances and represent incidence-based community patterns. Stress value is 0.12. Points represent individual samples, coloured by method (**A**) or tree species (**C**). Ellipses represent 95% confidence intervals. Euler diagrams (**B**, **D**) are based on incidence data and illustrate the overlap in fungal operational taxonomic units between the two methods (**B**) or across three tree species (**D**), highlighting both shared and unique OTUs. The area of each section is proportional to the number of detected OTUs. All visualisations are based on the rarefied dataset

When all OTUs were considered, regardless of their abundance, DNA and RNA metabarcoding revealed significantly different fungal communities associated with the same seed lots (Fig. 2A; F = 11.49, R^2^ = 0.25, *p* < 0.001). Of the total 872 OTUs identified in the rarefied dataset, only 100 (11%) were shared between the datasets, while around 13% and 75% were unique for the DNA and RNA dataset, respectively (Fig. 2B). The proportion of OTUs shared between the two metabarcoding methods further differed across tree species: approximately 7% of OTUs were shared in *P. sylvestris*, compared to 13% in *F. sylvatica* and 21% *in A. alba* (Supplementary Fig. 3A–C). Comparable results were obtained using the non-rarefied dataset (Supplementary Fig. 4A–E). Significant differences in the composition of dominant fungal communities—assessed by Morisita–Horn dissimilarity, which gives greater weight to OTUs with higher read counts—were also observed between DNA and RNA datasets (Supplementary Fig. 5A; F = 1.97, R^2^ = 0.05, *p* < 0.05). However, as indicated by R^2^ values, the relative importance of the method in explaining the differences in fungal community composition decreased from 25 to 5% when an abundance-weighted beta diversity measure was used suggesting that the differences between methods were largely driven by rare OTUs.

Fungal communities also differed among tree species, both when analysing the entire community (Fig. 2C–D; F = 2.84, R^2^ = 0.13, *p* < 0.001) and when focussing on dominant taxa (Supplementary Fig. 5B; F = 1.75, R^2^ = 0.28, *p* < 0.001). The proportion of variation explained by tree species increased from 13% to nearly 28% when switching from incidence-based to abundance-weighted metrics, respectively, as indicated by R^2^ values, suggesting that taxa with higher read counts were more host-specific than rare taxa.

Dispersions differed significantly among tree species (F = 3.86, df = 2, *p* < 0.05) and between methods (F = 13.79, df = 1, *p* < 0.01) for Sørensen dissimilarity, reflecting heterogeneous within-group variation. In contrast, for Morisita–Horn dissimilarity, no significant differences in dispersion were detected for either tree species (F = 1.63, df = 2, *p* = 0.233) or method (F = 0.006, df = 1, *p* = 0.947), indicating homogeneous dispersions.

### Taxonomic compositional differences between methods and tree species

Of the 1,557,227 fungal reads and 942 OTUs in the non-rarefied dataset, around 72% of reads and 64% of OTUs were assigned to 338 genera (Supplementary Table 2). While the proportions of reads and OTUs assigned to genera were consistent between the DNA and RNA datasets, they differed markedly in the number of detected genera (110 in DNA vs. 305 in RNA). Of these, 77 genera (23%) were shared, while 33 (10%) and 228 (67%) were unique to the DNA and RNA datasets, respectively. Across host species, 84% of reads were assigned to genera in *F. sylvatica*, compared to 73% in *A. alba* and 60% in *P. sylvestris*, with similar proportions recorded in both DNA and RNA datasets (Supplementary Table 3). These reads corresponded to 65–74% of OTUs, depending on the tree species, again with comparable patterns between metabarcoding approaches. Overall, the number of detected genera was two- to threefold higher in the DNA dataset than in the RNA dataset across all three species.

**Fig. 3:**
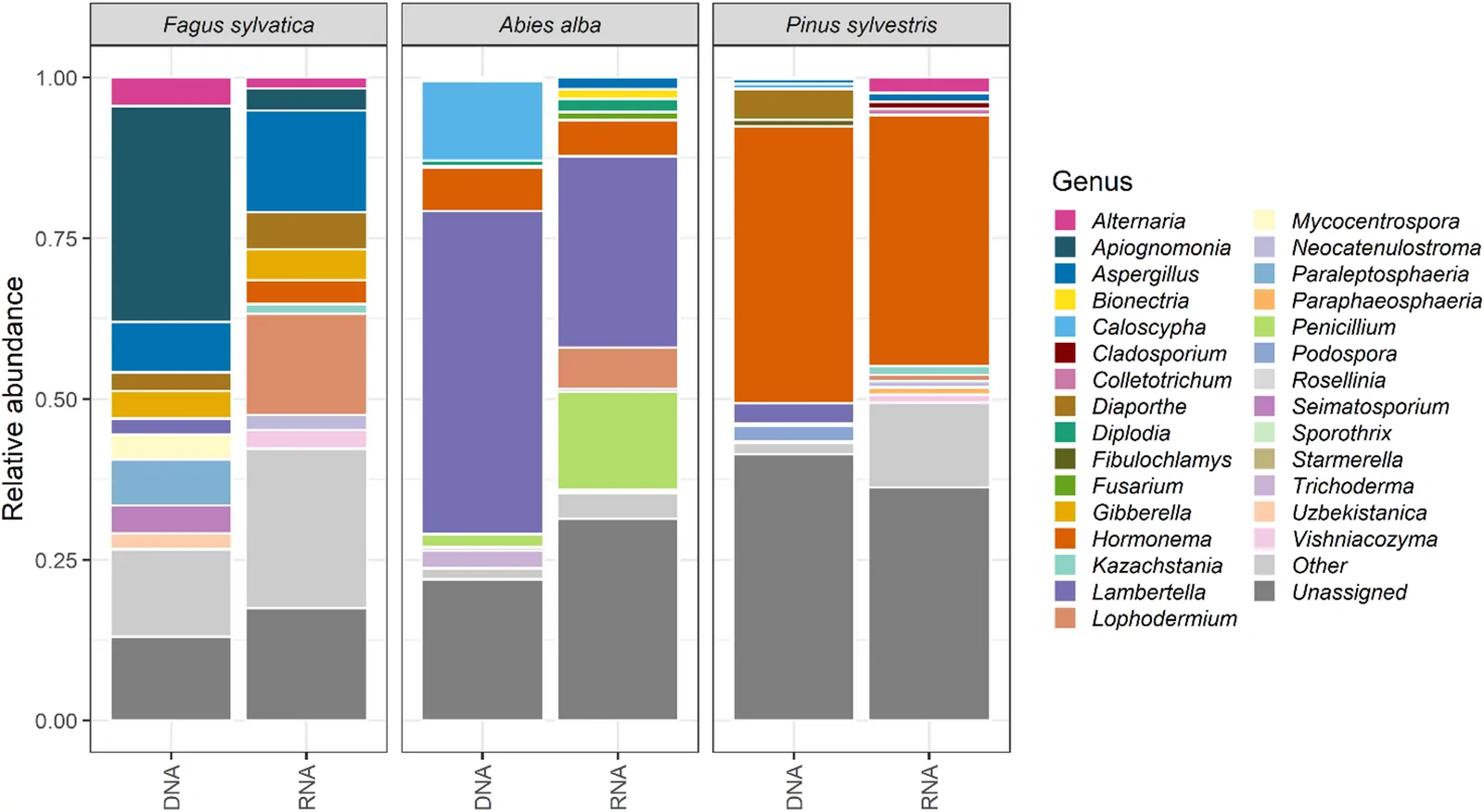
Dominant fungal genera in seeds of three tree species from DNA and RNA metabarcoding. Taxonomic profiles of dominant fungal communities in seeds of *Fagus sylvatica*, *Abies alba*, and *Pinus sylvestris*, based on DNA and RNA metabarcoding are shown. Bars show the relative abundance of the ten most abundant fungal genera per tree species and metabarcoding method. Reads assigned to genera outside the top ten are grouped under “Other,” while those not classified at genus level are labelled “Unassigned.” Calculations are based on non-rarefied dataset

Taxonomic composition of dominant genera (i.e., the ten most abundant genera per method and tree species) varied across host species and sequencing methods (Fig. 3). The only genus consistently identified among the top ten across all methods and tree species was *Aspergillus*. Among the top ten genera identified for each method and tree species, five were shared between the DNA and RNA datasets in *F. sylvatica* (i.e., *Alternaria*, *Apiognomonia*, *Aspergillus*, *Diaporthe*, *Gibberella*), five in *A. alba* (i.e., *Aspergillus*, *Diplodia*, *Hormonema*, *Lambertella*, *Penicillium*), and two in *P. sylvestris* (i.e., *Aspergillus*, *Hormonema*). These shared genera accounted for a high proportion of reads in each dataset (i.e., *F. sylvatica*: 53% in DNA and 32% in RNA; *A. alba*: 60% in DNA and 55% in RNA; *P. sylvestris*: 44% in DNA and 40% in RNA).

Indicator species analysis revealed significant, dataset-specific associations of genera with either DNA or RNA datasets (Supplementary Table 1). No genera were consistently associated with the DNA datasets across all three tree species, whereas *Kazachstania* and *Vishniacozyma* were associated with the RNA datasets, albeit with varying relative abundances. In *F. sylvatica*, three genera were associated with the DNA dataset and nine with the RNA dataset. Of these, three RNA-associated genera—*Lophodermium*, *Kazachstania* and *Vishniacozyma*—ranked among the top ten most abundant genera. In *A. alba*, ten genera were indicators of each dataset. Among the dominant genera, *Caloscypha* was strongly linked to DNA, while *Fusarium* and *Lophodermium* were linked to the RNA dataset. In *P. sylvestris*, ten genera were associated with the DNA dataset and six with the RNA dataset. Within the top ten genera, *Caloscypha*, *Podospora*, and *Lambertella* were DNA-associated, whereas *Colletotrichum*, *Kazachstania*, and *Vishniacozyma* were strongly associated with the RNA dataset. Additional genus-level associations were detected (Supplementary Table 1) but fell outside the top ten most abundant genera per method and species.

### Lifestyle compositional differences between methods and tree species

Of the total reads and OTUs in the non-rarified dataset (1,557,227 reads and 942 OTUs), a primary lifestyle could be assigned to 1,128,634 reads (74%) representing 596 OTUs (64%). Most fungal reads and OTUs belonged to saprotrophs (72% of reads and 59% of OTUs), followed by plant pathogens (25% of reads and 24% of OTUs), while mycorrhizal fungi, endophytes, and taxa categorized as “Other” each accounted for only a small fraction of the reads and OTUs (Supplementary Table 4). The proportion of reads and OTUs assigned to each lifestyle category was similar in the DNA and RNA datasets.

**Fig. 4:**
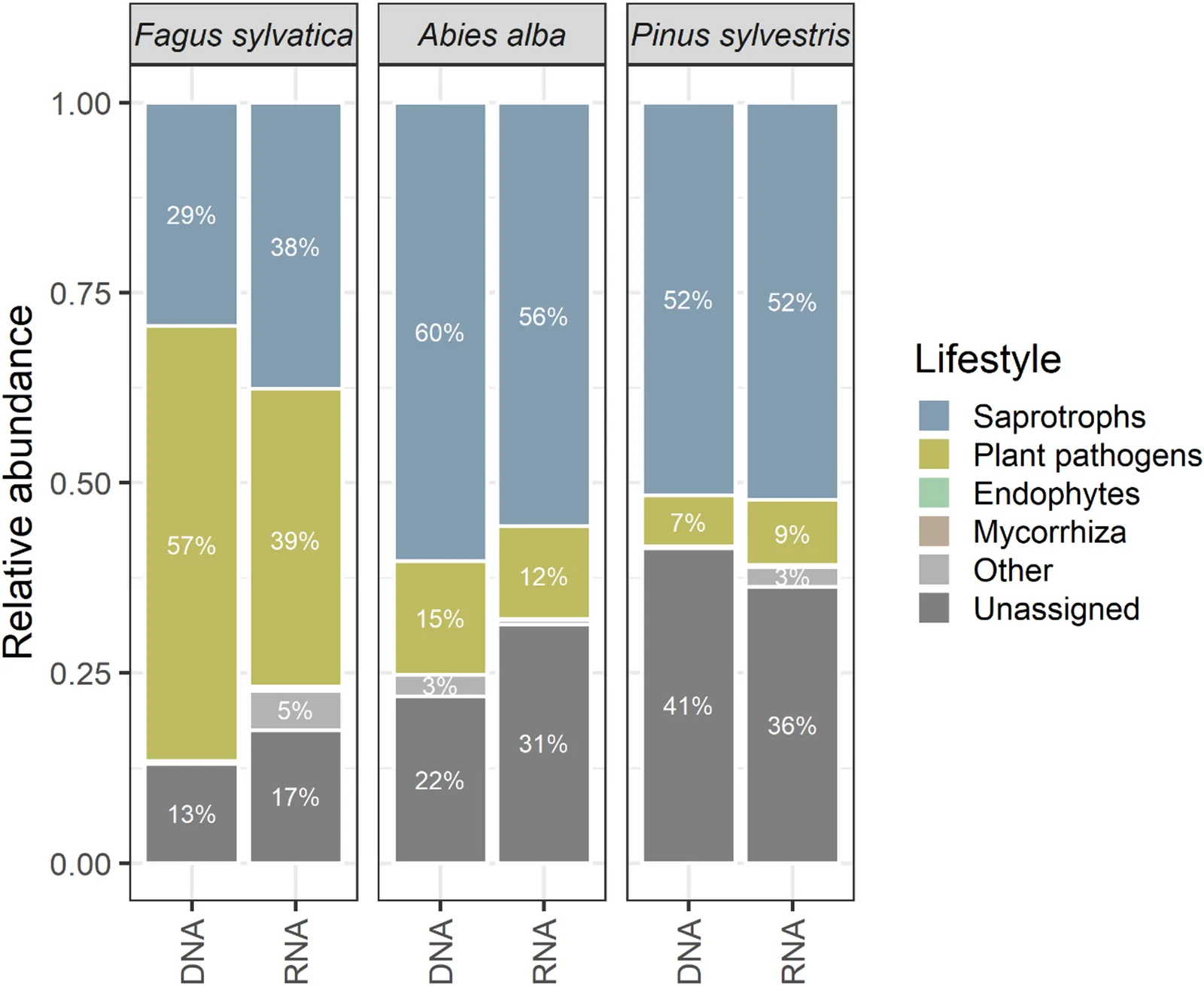
Fungal lifestyle composition in seeds of three tree species from DNA and RNA metabarcoding*.* Lifestyle profiles of fungal communities in seeds of *Fagus sylvatica*, *Abies alba*, and *Pinus sylvestris*, based on DNA and RNA metabarcoding are shown. Fungal lifestyles were assigned using FungalTraits database. Bars show the relative abundance of different fungal lifestyles per tree species and metabarcoding method. Percentages are not shown for lifestyles representing less than 1% of reads per method and tree species. Calculations are based on non-rarefied dataset

Approximately 85%, 75%, and 65% of reads, and 67%, 73% and 64% of OTUs from *F. sylvatica*, *A. alba*, and *P. sylvestris*, respectively, were classified into fungal lifestyles, with varying proportions between the two datasets (Fig. 4, Supplementary Table 5). In *F. sylvatica*, plant pathogens accounted for 57% of DNA reads and 39% of RNA reads, while saprotrophs were represented with 29% of DNA reads and 38% of RNA reads. Saprotrophs were the most abundant guild in *A. alba* (60% in DNA and 56% in RNA) and *P. sylvestris* (52% in each dataset). Plant pathogens constituted 15% and 12% of DNA and RNA reads in *A. alba* and 7% and 9% in *P. sylvestris* DNA and RNA dataset, respectively. In *F. sylvatica* and *A. alba*, mycorrhizal fungi (0.42% and 0.08% reads) and endophytes (0.12% and 0.04% reads) were exclusively detected in the RNA dataset. Unlike *F. sylvatica* and *A. alba*, *P. sylvestris* contained endophytes in both DNA and RNA datasets at similar proportions (0.04% and 0.05%, respectively). Mycorrhizal fungi in *P. sylvestris* were detected in both datasets but with only < 0.01% reads in the DNA compared to 0.21% reads in the RNA dataset.

### Comparison of metabarcoding datasets with traditional culturing data

**Fig. 5:**
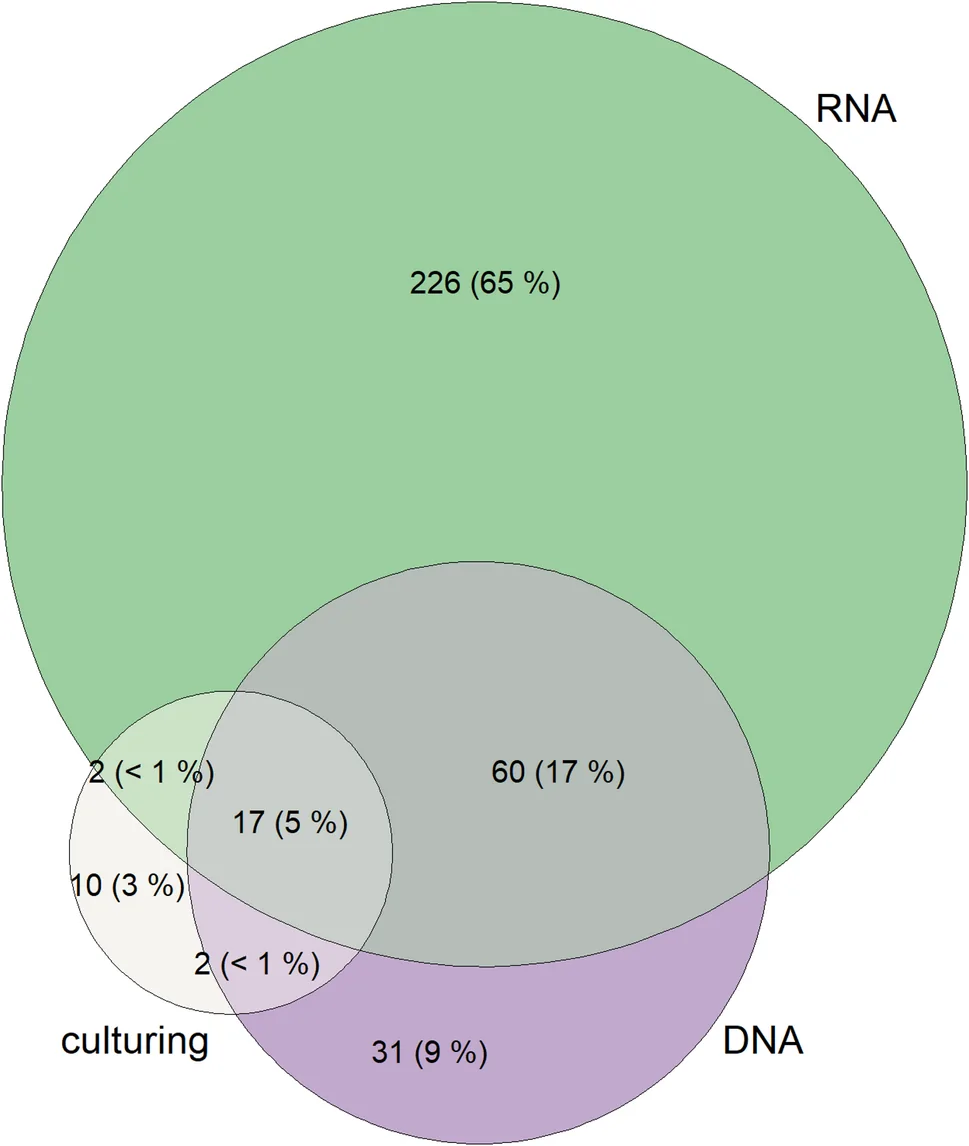
Overlap of fungal genera detected by culturing and DNA/RNA metabarcoding methods. Euler diagram illustrates the overlap in fungal genera detected by culturing and two metabarcoding methods (i.e., DNA and RNA), highlighting shared and unique OTUs. The diagram is based on the non-rarefied dataset, and the area of each section is proportional to the number of detected genera

A total of 1,553 fungal isolates were obtained across all seed samples and grouped into 203 morphotypes. From these, 203 representative isolates (i.e., one per morphotype) were selected for sequencing, of which 145 yielded good-quality sequences. Of those, 121 sequences were assigned to 31 distinct genera (Supplementary Table 6), compared to 110 and 305 genera identified by DNA and RNA metabarcoding, respectively. Of the 31 genera identified through culturing, ten (32%) were found exclusively by culturing. In contrast, 17 genera (55%) were also detected in both DNA and RNA datasets, while two additional genera were shared with each metabarcoding dataset individually. Although the overlap between culturing and metabarcoding was broadly similar—with most cultured genera appearing in both metabarcoding datasets—the RNA dataset contained a higher number of unique genera, whereas the DNA dataset included fewer unique taxa (Fig. 5). Similar patterns in the proportion of shared and unique genera across the three methods were observed for all three tree species (Table 2, Supplementary Fig. 6).

**Table 2:** Fungal genera cultured from three tree species and their detection in DNA/RNA metabarcoding datasets

Genus	Number of isolates	Number of morphotypes	Present in DNA dataset	Present in RNA dataset
***Fagus sylvatica***				
*Alternaria*	318	13	x	x
*Apiognomonia*	244	18	x	x
*Fusarium*	125	13	x	x
*Discosia*	57	5		
*Diaporthe*	42	4	x	x
*Epicoccum*	33	5		
*Cladosporium*	25	4		x
*Penicillium*	24	5	x	x
*Aureobasidium*	14	3	x	x
*Ceratobasidium*	10	1	x	x
*Oliveonia*	10	1	x	
*Clonostachys*	9	4		x
*Trichothecium*	9	1	x	x
*Seimatosporium*	8	2	x	x
*Xylaria*	8	2		
*Didymosphaeria*	6	1		
*Muriphaeosphaeria*	6	1	x	x
*Chaetomium*	5	1		x
*Biscogniauxia*	5	1		
*Radulidium*	5	1		
*Neosetophoma*	4	2	x	x
*Plagiostoma*	3	1		
*Boeremia*	3	1		
*Phacidium*	3	1	x	
unidentified	324	54		
***Abies alba***				
*Penicillium*	28	7	x	x
*Trichoderma*	27	2	x	x
*Sydowia*	25	2		
*Fusarium*	7	2	x	x
*Aspergillus*	6	3	x	x
*Mucor*	2	1		
*Talaromyces*	1	1	x	x
*Akanthomyces*	1	1		
unidentified	134	25		
***Pinus sylvestris***				
*Penicillium*	5	5	x	x
*Fusarium*	5	1	x	x
*Boeremia*	4	2		
*Coniochaeta*	3	1		x
*Aspergillus*	1	1	x	x
*Sydowia*	1	1		
unidentified	3	3		

Fungal genera detected in the culturing data of the three tree species (i.e., *F. sylvatica*, *A. alba*, *P. sylvestris)* are listed. Number of isolates belonging to each genus and number of morphotypes those isolates belong to is indicated, as well as the information about if the genera were detected in either of the two metabarcoding datasets (i.e., DNA, RNA)

## Discussion

Traditional culturing methods reveal limited fungal diversity, are resource-intensive, and have largely been replaced by high-throughput metabarcoding approaches in studies where living cultures are not required. Metabarcoding studies are predominantly DNA-based, which does not allow differentiation between metabolically active and inactive taxa, including dormant or dead organisms. In contrast, RNA metabarcoding targets living, metabolically active organisms, potentially providing a more ecologically relevant snapshot of fungal communities compared to DNA metabarcoding. RNA metabarcoding has shown potential for studying active environmental microbiota [18, 25, 28, 59], yet the method has rarely been applied for characterising microorganisms associated with plants. Nonetheless, some examples exist. Nguyen et al. [30] studied the effects of tree species richness on the foliar microbiota of Norway spruce (*Picea abies* (L.) H. Karst.), with both DNA- and RNA-based metabarcoding revealing largely similar microbial communities and inconsistent responses to tree species richness. Nash et al. [60] showed that RNA-based metabarcoding captures the temporally dynamic**,** metabolically active subset of the quaking aspen (*Populus tremuloides* Michx.) root microbiota, while Nerva et al. [61] applied an RNA approach to assess bacterial communities in grapevine (*Vitis vinifera* L.) wood of different health status. Given its high potential for ecological and phytosanitary monitoring, it is crucial to further evaluate the RNA metabarcoding against other commonly applied methods for studying plant-associated fungi before it can be widely implemented.

In this study, we compared incidence-based and abundance-weighted patterns of seed-borne fungal diversity and community composition between DNA and RNA metabarcoding methods and among tree species. Additionally, we assessed the overlap between fungal genera identified through culturing and those detected by each metabarcoding method. Our results show that dominant fungal communities are shaped primarily by host species identity, with the metabarcoding approach playing a comparatively minor role. Differences between metabarcoding approaches were largely driven by rare OTUs detected in the RNA dataset, whose biological relevance remains uncertain given methodological differences between workflows. Notably, both approaches recovered similar cultured genera, which likely represent abundant and metabolically active taxa.

### Fungal diversity and community composition across metabarcoding and culturing datasets

Contrary to our hypothesis, RNA metabarcoding revealed a higher number of fungal OTUs than DNA metabarcoding, including many unique OTUs, resulting in highly divergent community profiles when fungal incidence was analysed. However, these differences may partially reflect variation in group dispersion, and not the differences in group centroids, as indicated by a significant dispersion effect. While abundance-weighted alpha diversity was also lower in the DNA than in the RNA dataset, the divergence between DNA- and RNA-based community profiles decreased when abundance-weighted metrics were applied. Among the most abundant genera identified per tree species and method, about half were shared between the DNA and RNA datasets in two out of three tree species. This indicates that many dominant fungi are likely metabolically active, whereas discrepancies between methods are more strongly associated with rare taxa.

The higher diversity observed in the RNA dataset relative to DNA dataset is unlikely to reflect biological differences between total and metabolically active communities alone but is more plausibly explained by methodological differences between workflows. In particular, differences in subsampling and nucleic acid extraction procedures likely represent major sources of bias. Subsampling strategies differed between workflows—a single DNA extraction was performed per sample, whereas RNA was extracted in duplicates and pooled to obtain sufficient input for reverse transcription. This increased sampling effort for RNA likely inflated observed diversity and influenced community composition independently of biological differences, as previously shown for seed mycobiota of *P. sylvestris* [62]. Although abundance-weighted diversity metrics were used to partially account for variation in sampling effort [63], such adjustments cannot fully compensate for differences introduced during earlier stages of data generation. In addition, DNA and RNA were obtained using different extraction kits, and differences in extraction protocols can significantly influence microbial community composition [64]. In future studies, this bias could be mitigated by using co-extraction protocols that simultaneously isolate DNA and RNA [65].

Furthermore, the reverse transcription step required to synthesize cDNA from RNA introduces biases such as uneven abundances of correctly transcribed molecules. The reverse transcription can also introduce artefacts due to incorrect primer binding or unpredictable reverse transcriptase behaviour [66]. These factors likely contributed to discrepancies between fungal community profiles derived from DNA and RNA. Additional bias may occur during PCR amplification of the ITS region originating from both DNA and cDNA, and library preparation, including primer-template mismatches and selective amplification of certain taxa [67]. Finally, DNA and RNA libraries were sequenced on separate runs, which may have introduced further variation, although, this effect is likely less pronounced than the other previously mentioned methodological sources of bias [64].

We found no evidence supporting our hypothesis that cultured fungi would more closely resemble the RNA-derived community than the DNA-derived one. Approximately 50% of cultured genera were detected by both metabarcoding approaches, while an additional 6% was uniquely recovered by either DNA- or RNA-based metabarcoding. These results suggest that both metabolic activity and abundance influence the success of isolation of the fungus. If culturing primarily captured metabolically active taxa, a stronger overlap with the RNA dataset—representing rare but active fungi—would have been expected. Instead, our culturing approach likely favoured abundant and competitive fungi [8]. In addition, culturing may also recover dormant but viable propagules (e.g., spores or sclerotia), which are detectable by DNA but may be underrepresented in RNA-based metabarcoding. Alternative strategies such as dilution-to-extinction culturing [68] or use of different media [69] could help recover a greater diversity of rare yet cultivable taxa and potentially increase overlap with RNA-based profiles. As expected, culturing recovered substantially lower diversity than metabarcoding, reflecting the well-known difficulty of growing many fungal taxa under standard laboratory conditions [8]. Moreover, taxa detected only by culturing may reflect stochastic recovery or primer bias between methods. As isolates are critical for species description, functional studies, and phenotypic assays, continued improvement of culture-dependent techniques remains essential. Developing more sensitive and high-throughput culturing approaches will be key to bridging molecular and culture-based views on fungal diversity.

### Taxonomic and functional fungal community composition across metabarcoding datasets and tree species

Tree species identity was a major driver of the community composition of dominant fungi, explaining approximately 30% of the overall observed variation. The high host specificity of seed-borne fungi observed in our study aligns with findings from both woody and non-woody plants [70, 71]. Although, several dominant fungal genera were associated with already known hosts, not all of them were previously known to occur in seeds. For example, the genus *Apiognomonia* was the most abundant in *F. sylvatica* seed, likely corresponding to *A. errabunda*, a common endophyte of *F. sylvatica* known to cause anthracnose under wet spring conditions or following insect damage [72], and was also identified in the culturing dataset. *Abies alba* seeds were dominated by OTUs assigned to the genus *Lambertella* which has been previously found in *A. koreana* needles [73]. *Pinus sylvestris* seeds were dominated by the genus *Hormonema*, likely corresponding to *Sydowia polyspora* (i.e., old name is *H. dematioides*), a well-known endophyte and opportunistic pathogen in conifers and pre-emergent seed pathogen in *P. ponderosa* [6, 74], which was also identified in the culturing dataset.

Some fungal genera observed in seeds of specific tree species were found to be strongly associated with one of the metabarcoding datasets. For example, the genus *Caloscypha*, likely represented by the species *C. fulgens*—a well-known conifer seed pathogen [75] was strongly associated with the *A. alba* and *P. sylvestris* DNA dataset. The genus *Lophodermium* showed strong association with the RNA data of *F. sylvatica* and *A. alba* and was represented by multiple OTUs identified to several species known as needle endophytes or pathogens of conifers [76]. Association of *Lophodermium* with *F. sylvatica* may stem from environmental or laboratory contamination. In *P. sylvestris*, *Colletotrichum* was strongly associated with the RNA dataset. Some *Colletotrichum* species are known pathogens of pines, although their occurrence in conifers is less frequent than in broadleaf hosts [77]. These results highlight the value of combining DNA and RNA-based metabarcoding to uncover both inactive and active fungal associations, some of which may have implication for tree health.

Similar to findings by Mittelstrass et al. [3] most fungal reads in our study were assigned to saprotrophs and plant pathogens, which appeared in similar proportions across DNA and RNA metabarcoding datasets of all three host species. This suggests that these functional groups are not only abundant but also metabolically active in dormant tree seeds. Although dormant seeds may not seem like suitable habitats—given that saprotrophs rely on dead organic matter and plant pathogens require a susceptible host—research suggests that many potentially saprotrophic or pathogenic taxa can persist in asymptomatic plant tissues as endophytes (i.e., asymptomatic, commensal or weakly mutualistic inhabitants; [46]) and shift to saprotrophic or pathogenic lifestyle under favourable conditions [78]. Mycorrhizal fungi and endophytes were detected almost exclusively in RNA datasets. The sporadic occurrence across seed lots and low abundance of mycorrhizal fungi in seeds, which may likely be due to contamination, supports the notion that their seed-transmission is uncommon in trees [70, 79]. Low numbers of endophytic taxa are likely associated with their underrepresentation in databases such as the FungalTraits [46], underscoring the need for more targeted research into their diversity and function.

## Conclusions

Our results show that DNA and RNA metabarcoding provide complementary insights into dominant fungal communities and have valuable potential for high-throughput ecological and biosecurity monitoring. DNA metabarcoding has already been widely adopted for assessing biodiversity of different organisms in both environmental and host-associated samples. Its ability to detect living and non-living taxa provides a comprehensive perspective on total fungal community associated with the studied system. In contrast, RNA-based metabarcoding is still less commonly applied, despite its focus on metabolically active organisms which may enable real-time insights into ecosystem dynamics and functional responses to environmental change. Moreover, RNA metabarcoding might be particularly promising for biosecurity application where rapid detection and identification of living pests and pathogens is critical for decision making and timely intervention.

While our results show that most dominant taxa are captured by both methods, RNA metabarcoding revealed higher diversity, driven mainly by rare taxa. However, methodological differences between DNA and RNA workflows, as well as the lack of negative controls, limit direct comparability. Consequently, observed differences between DNA and RNA datasets are likely driven by methodological factors and cannot be attributed solely to biological differences. These discrepancies should therefore be interpreted cautiously. Future research using standardized protocols and appropriate controls will be essential to better resolve these methodological effects and improve confidence in rare taxon detection.

The inability of RNA approaches to detect metabolically inactive yet viable organisms represent a limitation as dormant pathogens may escape detection and later become active. This could be tackled with applying viability PCR (vPCR) using propidium or ethidium monoazide which enables selective suppression of DNA from membrane-compromised cells, allowing DNA-based assays to target only intact, viable fungi and thus reduce false positives from relic DNA [80, 81]. Nevertheless, vPCR also has important limitations. Its effectiveness can vary depending on cell wall structure and environmental matrix composition, and incomplete dye penetration or binding may lead to false negatives. Moreover, its applicability to mixed environmental samples such as those that need to be ground prior to DNA/RNA extraction, remains uncertain. If technical obstacles are overcome, performing DNA metabarcoding with and without vPCR pretreatment could help differentiate living from dead organisms. Combining this with RNA-based methods can further help identify the metabolically active members of the community, leading to more accurate ecological interpretations and risk assessments.

Looking ahead, advances in sequencing technologies and bioinformatics are expected to increase the sensitivity and resolution of metabarcoding methods, enabling more precise taxonomic identification and functional profiling. In the face of growing environmental pressures and accelerating species movement through trade, the combined use of DNA and RNA metabarcoding holds strong potential for ecosystem management and biosecurity, by revealing total and active communities. While culturing remains essential for studying fungal diversity, pathogenicity and functional traits, traditional approaches recover only a subset of the taxa detected by metabarcoding, typically the dominant and readily cultivable species. Future efforts should thus focus on developing more sensitive, high-throughput culturing techniques capable of capturing a broader spectrum of fungal diversity. Ultimately, integrating all three approaches—DNA- and RNA-based metabarcoding together with advanced culturing—offers the most comprehensive framework for studying fungal diversity and strengthening biosecurity, by capturing both active and inactive taxa as well as culturable and unculturable species.

## Supplementary Information

Below is the link to the electronic supplementary material.

Supplementary Material 1

## Data Availability

Raw metabarcoding sequence reads have been deposited in the European Nucleotide Archive (ENA) at EMBL-EBI under accession number PRJEB96213. ITS barcode sequences generated from cultured specimens have been deposited in GenBank under accession numbers PX736467–PX736489, PX740711–PX740835, and PX745229–PX745242. All ITS sequences used in this study, together with their corresponding accession numbers, are provided in Supplementary Table 6.
