# Supplementary material for: DNA and RNA metabarcoding reveal shared dominant seed-borne fungi
Source: Environ Microbiome. 2026 Jul 3;21:112. doi: 10.1186/s40793-026-00923-6 (PMC13625387; doi:10.1186/s40793-026-00923-6)
Supplement: Supplementary file 1 [file 40793_2026_923_MOESM1_ESM.docx]

## Supplementary Tables

**Supplementary Table 1.** **Fungal genera significantly associated with DNA or RNA datasets for each of the three tree species.** The significance is assessed using Indicator Species Analysis. The Indicator Value (IndVal; range 0–1) reflects the strength of association between each genus and a given dataset. Higher IndVal scores indicate stronger indicators. Only statistically significant associations (p < 0.05) are shown, along with FDR-adjusted p-values. Genera ranked among the top ten most abundant within each method × tree species combination are highlighted in bold.

| **Dataset** | **Fungal genus** | **IndVal** | **p** | **p adjusted** |
| --- | --- | --- | --- | --- |
| *Fagus sylvatica* | | | | |
| DNA | *Muriphaeosphaeria* | 0.961 | 0.011 | 0.243 |
| DNA | *Oliveonia* | 0.913 | 0.014 | 0.243 |
| DNA | *Phoma* | 0.884 | 0.043 | 0.442 |
| RNA | *Botryosphaeria* | 1.000 | 0.003 | 0.119 |
| **RNA** | ***Lophodermium*** | **1.000** | **0.003** | **0.119** |
| **RNA** | ***Kazachstania*** | **1.000** | **0.003** | **0.119** |
| **RNA** | ***Vishniacozyma*** | **0.999** | **0.003** | **0.119** |
| RNA | *Bionectria* | 0.963 | 0.009 | 0.243 |
| RNA | *Colletotrichum* | 0.913 | 0.015 | 0.243 |
| RNA | *Cutaneotrichosporon* | 0.913 | 0.015 | 0.243 |
| RNA | *Diutina* | 0.913 | 0.015 | 0.243 |
| RNA | *Saccharomyces* | 0.913 | 0.015 | 0.243 |
| *Abies alba* | | | | |
| DNA | *Dictyochaeta* | 1.000 | 0.007 | 0.057 |
| DNA | *Paraleptosphaeria* | 1.000 | 0.007 | 0.057 |
| DNA | *Starmerella* | 1.000 | 0.007 | 0.057 |
| DNA | *Kodamaea* | 1.000 | 0.007 | 0.057 |
| DNA | *Phoma* | 1.000 | 0.007 | 0.057 |
| **DNA** | ***Caloscypha*** | **1.000** | **0.007** | **0.057** |
| DNA | *Mycocentrospora* | 0.964 | 0.007 | 0.057 |
| DNA | *Apiognomonia* | 0.950 | 0.007 | 0.057 |
| DNA | *Uzbekistanica* | 0.894 | 0.042 | 0.282 |
| DNA | *Podospora* | 0.894 | 0.049 | 0.282 |
| RNA | *Vishniacozyma* | 1.000 | 0.007 | 0.057 |
| RNA | *Neocatenulostroma* | 1.000 | 0.007 | 0.057 |
| **RNA** | ***Lophodermium*** | **1.000** | **0.007** | **0.057** |
| RNA | *Kazachstania* | 1.000 | 0.007 | 0.057 |
| RNA | *Pleonectria* | 1.000 | 0.007 | 0.057 |
| **RNA** | ***Fusarium*** | **1.000** | **0.007** | **0.057** |
| RNA | *Diutina* | 0.894 | 0.047 | 0.282 |
| RNA | *Mortierella* | 0.894 | 0.047 | 0.282 |
| RNA | *Cutaneotrichosporon* | 0.894 | 0.047 | 0.282 |
| RNA | *Metschnikowia* | 0.894 | 0.049 | 0.282 |
| *Pinus sylvestris* | | | | |
| DNA | *Paraleptosphaeria* | 1.000 | 0.008 | 0.147 |
| DNA | *Starmerella* | 1.000 | 0.008 | 0.147 |
| DNA | *Kodamaea* | 1.000 | 0.008 | 0.147 |
| DNA | *Phoma* | 1.000 | 0.008 | 0.147 |
| DNA | *Mycocentrospora* | 1.000 | 0.008 | 0.147 |
| **DNA** | ***Podospora*** | **0.998** | **0.008** | **0.147** |
| **DNA** | ***Caloscypha*** | **0.998** | **0.008** | **0.147** |
| **DNA** | ***Lambertella*** | **0.997** | **0.008** | **0.147** |
| DNA | *Apiognomonia* | 0.938 | 0.008 | 0.147 |
| DNA | *Dictyochaeta* | 0.894 | 0.046 | 0.613 |
| **RNA** | ***Colletotrichum*** | **1.000** | **0.008** | **0.147** |
| RNA | *Botryosphaeria* | 1.000 | 0.008 | 0.147 |
| **RNA** | ***Kazachstania*** | **1.000** | **0.008** | **0.147** |
| RNA | *Saccharomycopsis* | 1.000 | 0.008 | 0.147 |
| **RNA** | ***Vishniacozyma*** | **0.998** | **0.008** | **0.147** |
| RNA | *Cephalotrichum* | 0.894 | 0.047 | 0.613 |

**Supplementary Table 2.** **Fungal reads and OTUs assigned to different taxonomic levels.** The table also shows the number of distinct (unique) taxa identified within each level. Values are shown for DNA and RNA dataset separately, and for all data together. The values are expressed as both absolute counts and percentages of the total non-rarefied dataset. Total numbers of reads and OTUs are indicated in the Table 1.

|  | **DNA** | | | **RNA** | | | **All data** | | |
| --- | --- | --- | --- | --- | --- | --- | --- | --- | --- |
| **Taxonomic level** | **Reads** | **OTUs** | **Unique taxa** | **Reads** | **OTUs** | **Unique taxa** | **Reads** | **OTUs** | **Unique taxa** |
| **Class** | 762,771 (100%) | 213 (86%) | 15 | 777,500 (98%) | 710 (89%) | 36 | 1,540,271 (99%) | 820 (87%) | 37 |
| **Order** | 762,667 (100%) | 207 (84%) | 37 | 772,642 (97%) | 676 (84%) | 92 | 1,535,309 (99%) | 781 (83%) | 95 |
| **Family** | 759,549 (100%) | 187 (76%) | 77 | 759,484 (96%) | 583 (73%) | 191 | 1,519,033 (92%) | 674 (72%) | 203 |
| **Genus** | 563,572 (74%) | 168 (68%) | 110 | 565,143 (71%) | 519 (65%) | 305 | 1,128,715 (72%) | 600 (64%) | 338 |

**Supplementary Table 3.** **Fungal reads and OTUs assigned to genera*.*** Number of fungal reads and OTUs assigned to genera in samples belonging to the three tree species (i.e., *F. sylvatica*, *A. alba*, *P. sylvestris*; numbers of seed lots are indicated in brackets) are indicated. Numbers are shown for the DNA and RNA datasets and for the combined dataset (i.e., all data) considering all fungal reads. The values are expressed as both absolute counts and percentages of the total dataset. Total number of reads and OTUs are indicated in Table 1. Number of unique genera identified in DNA and RNA datasets across three tree species is also indicated.

|  | **Reads** | | | **OTUs** | | | **Genera** | | |
| --- | --- | --- | --- | --- | --- | --- | --- | --- | --- |
| **Tree species** | **DNA** | **RNA** | **All data** | **DNA** | **RNA** | **All data** | **DNA** | **RNA** | **All data** |
| ***F. sylvatica* (n = 6)** | 91,682 | 146,405 | 238,087 | 80 | 244 | 281 | 61 | 170 | 196 |
|  | 87% | 83% | 84% | 74% | 68% | 67% |  |  |  |
| ***A. alba* (n = 5)** | 347,149 | 358,907 | 706,056 | 76 | 171 | 204 | 49 | 114 | 133 |
|  | 78% | 69% | 73% | 82% | 74% | 74% |  |  |  |
| ***P. sylvestris* (n = 5)** | 124,741 | 59,831 | 184,572 | 104 | 340 | 407 | 81 | 223 | 266 |
|  | 59% | 64% | 60% | 68% | 66% | 65% |  |  |  |
| **Total** | 563,572 | 565,143 | 1,128,715 | 168 | 519 | 600 | 110 | 305 | 338 |
|  | 74% | 71% | 72% | 68% | 65% | 64% |  |  |  |

**Supplementary Table 4.** **Fungal reads and OTUs assigned to different lifestyle categories across metabarcoding datasets.** The values are expressed as both absolute counts and percentages of the total dataset (i.e., containing 1,128,634 reads and 596 OTUs that were assigned to a lifestyle). Lifestyle assignments are based on the “primary lifestyle” column from the *FungalTraits* database. Values are presented separately for DNA and RNA datasets, as well as combined totals across all samples.

|  | **Reads** | | | **OTUs** | | |
| --- | --- | --- | --- | --- | --- | --- |
| **Lifestyle** | **DNA** | **RNA** | **All data** | **DNA** | **RNA** | **All data** |
| **Saprotrophs** | 408,977 (73%) | 406,805 (72%) | 815,782 (72%) | 106 (63%) | 294 (57%) | 349 (59%) |
| **Plant pathogens** | 141,052 (25%) | 141,638 (25%) | 282,690 (25%) | 50 (30%) | 125 (24%) | 143 (24%) |
| **Mycorrhiza** | 3 (<1%) | 1,333 (<1%) | 1,1336 (<1%) | 1  (1%) | 20 (4%) | 21 (4%) |
| **Endophytes** | 85 (<1%) | 501 (<1%) | 586 (<1%) | 1 (1%) | 6 (1%) | 6 (1%) |
| **Other** | 13,455 (2%) | 14,785 (3%) | 28,240 (3%) | 10 (6%) | 70 (14%) | 77 (13%) |

**Supplementary Table 5.** **Fungal reads and OTUs assigned to different lifestyle categories across tree species and metabarcoding datasets*.*** Number of fungal reads (A) and OTUs (B) assigned to different lifestyle categories in samples belonging to the three tree species (i.e., *F. sylvatica*, *A. alba*, *P. sylvestris*; numbers of seed lots are indicated in brackets) are indicated. Numbers are shown for the DNA and RNA datasets considering all fungal reads. The values are expressed as both absolute counts and percentages of the total dataset. Total number of reads and OTUs are indicated in Table 1.

| A | |  |  |  |  |  |  |
| --- | --- | --- | --- | --- | --- | --- | --- |
|  |  | **Reads** | | | | | |
|  |  | **Plant pathogens** | **Saprotrophs** | **Endophytes** | **Mycorrhiza** | **Other** | **Unassigned** |
| ***F. sylvatica***  **(n = 6)** | **DNA** | 60,347 | 30,903 | 0 | 0 | 432 | 13,714 |
|  |  | 57.26% | 29.32% | 0% | 0% | 0.41% | 13.01% |
|  | **RNA** | 69,463 | 66,791 | 217 | 740 | 9,194 | 31,015 |
|  |  | 39.15% | 37.65% | 0.12% | 0.42% | 5.18% | 17.48% |
| ***A. alba***  **(n = 5)** | **DNA** | 66,532 | 268,151 | 0 | 0 | 12,466 | 97,685 |
|  |  | 14.96% | 60.28% | 0% | 0% | 2.80% | 0.014% |
|  | **RNA** | 64,083 | 291,014 | 233 | 392 | 3,134 | 164,025 |
|  |  | 12.26% | 55.66% | 0.04% | 0.075% | 0.60% | 31.37% |
| ***P. sylvestris***  **(n = 5)** | **DNA** | 14,173 | 109,923 | 85 | 3 | 557 | 88,079 |
|  |  | 6.66% | 51.65% | 0.04% | 0.001% | 0.26% | 41.39% |
|  | **RNA** | 8,092 | 49,000 | 51 | 201 | 2,457 | 34,075 |
|  |  | 8.62% | 52.20% | 0.05% | 0.21% | 2.62% | 36.30% |
| B | |  |  |  |  |  |  |
|  |  | **OTUs** | | | | | |
|  |  | **Plant pathogens** | **Saprotrophs** | **Endophytes** | **Mycorrhiza** | **Other** | **Unassigned** |
| ***F. sylvatica***  **(n = 6)** | **DNA** | 34 | 41 | 0 | 0 | 5 | 28 |
|  |  | 31.48% | 37.96% | 0% | 0% | 4.63% | 25.93% |
|  | **RNA** | 73 | 126 | 2 | 12 | 31 | 117 |
|  |  | 20.22% | 34.90% | 0.55% | 3.32% | 8.59% | 32.41% |
| ***A. alba***  **(n = 5)** | **DNA** | 22 | 48 | 0 | 0 | 6 | 17 |
|  |  | 23.66% | 51.61% | 0% | 0% | 6.45% | 18.28% |
|  | **RNA** | 52 | 94 | 2 | 3 | 19 | 62 |
|  |  | 22.41% | 40.52% | 0.86% | 1.29% | 8.19% | 26.72% |
| ***P. sylvestris***  **(n = 5)** | **DNA** | 27 | 70 | 1 | 1 | 5 | 48 |
|  |  | 17.76% | 46.05% | 0.66% | 0.66% | 3.29% | 31.58% |
|  | **RNA** | 70 | 200 | 3 | 9 | 54 | 183 |
|  |  | 13.49% | 38.55% | 0.58% | 1.73% | 10.41% | 35.26% |
|  |  |  |  |  |  |  |  |

**Supplementary Table 6.** **ITS sequences obtained from cultured isolates selected as representatives of fungal morphogroups.** For each sequence, the sequence ID, morphogroup ID, and the number of isolates within the corresponding morphogroup are indicated, along with the organism’s taxonomic identity, the host’s taxonomic identity, and the ENA accession number under which the sequence is deposited.

| **Sequence ID** | **Morphogroup ID** | **Number of isolates** | **Organism ID** | **Host** | **Accession number** |
| --- | --- | --- | --- | --- | --- |
| AA5 | AA_M3 | 3 | *Penicillium sp.* | *Abies alba* | PX736467 |
| AA7 | AA_M4 | 5 | *Fusarium sp.* | *Abies alba* | PX736468 |
| AA9 | AA_M5 | 6 | *Trichoderma sp.* | *Abies alba* | PX736470 |
| AA11 | AA_M10 | 21 | *Trichoderma sp.* | *Abies alba* | PX736472 |
| AA16 | AA_M8 | 22 | *Sydowia polyspora* | *Abies alba* | PX736474 |
| AA18 | AA_M7 | 1 | *Akanthomyces sp.* | *Abies alba* | PX736475 |
| AA19 | AA_M6 | 2 | *Fusarium sp.* | *Abies alba* | PX736476 |
| AA23 | AA_M12 | 2 | *Mucor racemosus* | *Abies alba* | PX736477 |
| AA28 | AA_M16 | 3 | *Aspergillus sp.* | *Abies alba* | PX736479 |
| AA32 | AA_M20 | 2 | *Aspergillus sp.* | *Abies alba* | PX736480 |
| AA33 | AA_M21 | 1 | *Aspergillus sp.* | *Abies alba* | PX736481 |
| AA34 | AA_M22 | 9 | *Penicillium sp.* | *Abies alba* | PX736482 |
| AA36 | AA_M24 | 9 | *Penicillium sp.* | *Abies alba* | PX736483 |
| AA42 | AA_M27 | 1 | *Talaromyces sp.* | *Abies alba* | PX736484 |
| AA73 | AA_M45 | 2 | *Penicillium sp.* | *Abies alba* | PX736485 |
| AA79 | AA_M34 | 3 | *Sydowia polyspora* | *Abies alba* | PX736486 |
| AA83 | AA_M58 | 1 | *Penicillium sp.* | *Abies alba* | PX736487 |
| AA84 | AA_M63 | 3 | *Penicillium sp.* | *Abies alba* | PX736488 |
| AA90 | AA_M81 | 1 | *Penicillium sp.* | *Abies alba* | PX736489 |
| PS4 | PS_M1 | 5 | *Fusarium sp.* | *Pinus sylvestris* | PX745229 |
| PS9 | PS_M2 | 3 | *Coniochaeta sp.* | *Pinus sylvestris* | PX745230 |
| PS17 | PS_M12 | 1 | *Penicillium sp.* | *Pinus sylvestris* | PX745232 |
| PS18 | PS_M13 | 1 | *Penicillium sp.* | *Pinus sylvestris* | PX745233 |
| PS19 | PS_M8 | 3 | *Boeremia exigua* | *Pinus sylvestris* | PX745234 |
| PS23 | PS_M7 | 1 | *Sydowia polyspora* | *Pinus sylvestris* | PX745235 |
| PS26 | PS_M17 | 1 | *Boeremia sp.* | *Pinus sylvestris* | PX745237 |
| PS28 | PS_M19 | 1 | *Penicillium sp.* | *Pinus sylvestris* | PX745238 |
| PS32 | PS_M23 | 1 | *Aspergillus sp.* | *Pinus sylvestris* | PX745239 |
| PS33 | PS_M24 | 1 | *Cordycipitaceae sp.* | *Pinus sylvestris* | PX745240 |
| PS34 | PS_M25 | 1 | *Penicillium sp.* | *Pinus sylvestris* | PX745241 |
| PS35 | PS_M26 | 1 | *Penicillium sp.* | *Pinus sylvestris* | PX745242 |
| FS1 | FS_M1 | 155 | *Alternaria sp.* | *Fagus sylvatica* | PX740711 |
| FS3 | FS_M2 | 57 | *Pleosporales sp.* | *Fagus sylvatica* | PX740713 |
| FS5 | FS_M3 | 4 | *Pleosporales sp.* | *Fagus sylvatica* | PX740715 |
| FS7 | FS_M4 | 24 | *Fusarium sp.* | *Fagus sylvatica* | PX740717 |
| FS9 | FS_M5 | 9 | *Fusarium sp.* | *Fagus sylvatica* | PX740719 |
| FS11 | FS_M6 | 8 | *Fusarium sp.* | *Fagus sylvatica* | PX740721 |
| FS13 | FS_M7 | 2 | *Fusarium sp.* | *Fagus sylvatica* | PX740723 |
| FS15 | FS_M8 | 30 | *Fusarium sp.* | *Fagus sylvatica* | PX740725 |
| FS17 | FS_M9 | 13 | *Fusarium sp.* | *Fagus sylvatica* | PX740727 |
| FS19 | FS_M10 | 2 | *Nectriaceae sp.* | *Fagus sylvatica* | PX740729 |
| FS21 | FS_M11 | 2 | *Fusarium sp.* | *Fagus sylvatica* | PX740731 |
| FS23 | FS_M12 | 1 | *Fusarium sp.* | *Fagus sylvatica* | PX740733 |
| FS24 | FS_M13 | 16 | *Alternaria sp.* | *Fagus sylvatica* | PX740734 |
| FS26 | FS_M14 | 6 | *Didymosphaeria variabile* | *Fagus sylvatica* | PX740735 |
| FS28 | FS_M15 | 7 | *Discosia sp.* | *Fagus sylvatica* | PX740736 |
| FS30 | FS_M16 | 26 | *Alternaria sp.* | *Fagus sylvatica* | PX740737 |
| FS32 | FS_M17 | 9 | *Alternaria sp.* | *Fagus sylvatica* | PX740738 |
| FS34 | FS_M18 | 14 | *Discosia sp.* | *Fagus sylvatica* | PX740739 |
| FS36 | FS_M19 | 7 | *Discosia sp.* | *Fagus sylvatica* | PX740740 |
| FS38 | FS_M20 | 5 | *Alternaria sp.* | *Fagus sylvatica* | PX740741 |
| FS40 | FS_M21 | 5 | *Alternaria sp.* | *Fagus sylvatica* | PX740742 |
| FS42 | FS_M22 | 10 | *Discosia sp.* | *Fagus sylvatica* | PX740743 |
| FS44 | FS_M23 | 19 | *Discosia sp.* | *Fagus sylvatica* | PX740744 |
| FS46 | FS_M24 | 15 | *Didymellaceae sp.* | *Fagus sylvatica* | PX740745 |
| FS48 | FS_M25 | 24 | *Didymellaceae sp.* | *Fagus sylvatica* | PX740746 |
| FS52 | FS_M27 | 53 | *Chaetosphaeriaceae sp.* | *Fagus sylvatica* | PX740747 |
| FS54 | FS_M28 | 14 | *Chaetosphaeriaceae sp.* | *Fagus sylvatica* | PX740748 |
| FS56 | FS_M29 | 17 | *Diaporthe sp.* | *Fagus sylvatica* | PX740749 |
| FS58 | FS_M30 | 20 | *Diaporthe sp.* | *Fagus sylvatica* | PX740750 |
| FS60 | FS_M31 | 16 | *Epicoccum sp.* | *Fagus sylvatica* | PX740751 |
| FS62 | FS_M32 | 12 | *Epicoccum sp.* | *Fagus sylvatica* | PX740752 |
| FS66 | FS_M35 | 89 | *Apiognomonia errabunda* | *Fagus sylvatica* | PX740753 |
| FS70 | FS_M34 | 2 | *Epicoccum sp.* | *Fagus sylvatica* | PX740754 |
| FS72 | FS_M36 | 23 | *Apiognomonia errabunda* | *Fagus sylvatica* | PX740755 |
| FS74 | FS_M38 | 26 | *Apiognomonia errabunda* | *Fagus sylvatica* | PX740756 |
| FS76 | FS_M39 | 23 | *Apiognomonia errabunda* | *Fagus sylvatica* | PX740757 |
| FS78 | FS_M40 | 19 | *Apiognomonia errabunda* | *Fagus sylvatica* | PX740758 |
| FS82 | FS_M42 | 5 | *Apiognomonia errabunda* | *Fagus sylvatica* | PX740759 |
| FS84 | FS_M43 | 6 | *Pleosporales sp.* | *Fagus sylvatica* | PX740760 |
| FS88 | FS_M45 | 12 | *Apiognomonia errabunda* | *Fagus sylvatica* | PX740761 |
| FS90 | FS_M46 | 10 | *Apiognomonia errabunda* | *Fagus sylvatica* | PX740762 |
| FS92 | FS_M47 | 4 | *Apiognomonia errabunda* | *Fagus sylvatica* | PX740763 |
| FS94 | FS_M48 | 2 | *Apiognomonia errabunda* | *Fagus sylvatica* | PX740764 |
| FS96 | FS_M49 | 5 | *Apiognomonia errabunda* | *Fagus sylvatica* | PX740765 |
| FS98 | FS_M50 | 3 | *Apiognomonia errabunda* | *Fagus sylvatica* | PX740766 |
| FS100 | FS_M51 | 5 | *Apiognomonia errabunda* | *Fagus sylvatica* | PX740767 |
| FS102 | FS_M52 | 8 | *Apiognomonia errabunda* | *Fagus sylvatica* | PX740768 |
| FS104 | FS_M53 | 9 | *Trichothecium roseum* | *Fagus sylvatica* | PX740769 |
| FS106 | FS_M54 | 10 | *Aureobasidium sp.* | *Fagus sylvatica* | PX740770 |
| FS108 | FS_M55 | 5 | *Didymellaceae sp.* | *Fagus sylvatica* | PX740771 |
| FS110 | FS_M56 | 3 | *Aureobasidium sp.* | *Fagus sylvatica* | PX740772 |
| FS112 | FS_M57 | 8 | *Alternaria sp.* | *Fagus sylvatica* | PX740773 |
| FS114 | FS_M58 | 56 | *Alternaria sp.* | *Fagus sylvatica* | PX740774 |
| FS116 | FS_M59 | 8 | *Alternaria sp.* | *Fagus sylvatica* | PX740775 |
| FS118 | FS_M60 | 14 | *Alternaria sp.* | *Fagus sylvatica* | PX740776 |
| FS120 | FS_M61 | 13 | *Fusarium sp.* | *Fagus sylvatica* | PX740777 |
| FS122 | FS_M62 | 3 | *Alternaria sp.* | *Fagus sylvatica* | PX740778 |
| FS125 | FS_M63 | 3 | *Xylaria sp.* | *Fagus sylvatica* | PX740779 |
| FS126 | FS_M64 | 10 | *Oliveonia pauxilla* | *Fagus sylvatica* | PX740780 |
| FS128 | FS_M65 | 5 | *Xylaria sp.* | *Fagus sylvatica* | PX740781 |
| FS130 | FS_M66 | 4 | *Fusarium sp.* | *Fagus sylvatica* | PX740782 |
| FS132 | FS_M67 | 10 | *Fusarium sp.* | *Fagus sylvatica* | PX740783 |
| FS134 | FS_M68 | 6 | *Seimatosporium sp.* | *Fagus sylvatica* | PX740784 |
| FS136 | FS_M69 | 5 | *Alternaria sp.* | *Fagus sylvatica* | PX740785 |
| FS138 | FS_M70 | 6 | *Muriphaeosphaeria viburni* | *Fagus sylvatica* | PX740786 |
| FS140 | FS_M71 | 4 | *Pleosporales sp.* | *Fagus sylvatica* | PX740787 |
| FS142 | FS_M72 | 10 | *Nectriaceae sp.* | *Fagus sylvatica* | PX740788 |
| FS145 | FS_M73 | 10 | *Ceratobasidium sp.* | *Fagus sylvatica* | PX740789 |
| FS146 | FS_M74 | 5 | *Biscogniauxia mediterranea* | *Fagus sylvatica* | PX740790 |
| FS148 | FS_M75 | 8 | *Diaporthaceae sp.* | *Fagus sylvatica* | PX740791 |
| FS150 | FS_M76 | 8 | *Alternaria sp.* | *Fagus sylvatica* | PX740792 |
| FS152 | FS_M77 | 4 | *Clonostachys sp.* | *Fagus sylvatica* | PX740793 |
| FS154 | FS_M78 | 7 | *Fusarium sp.* | *Fagus sylvatica* | PX740794 |
| FS156 | FS_M79 | 2 | *Neosetophoma sp.* | *Fagus sylvatica* | PX740795 |
| FS158 | FS_M80 | 6 | *Didymellaceae sp.* | *Fagus sylvatica* | PX740796 |
| FS160 | FS_M81 | 2 | *Neosetophoma sp.* | *Fagus sylvatica* | PX740797 |
| FS162 | FS_M83 | 5 | *Chaetomium sp.* | *Fagus sylvatica* | PX740798 |
| FS164 | FS_M84 | 1 | *Aureobasidium sp.* | *Fagus sylvatica* | PX740799 |
| FS170 | FS_M86 | 4 | *Nectriaceae sp.* | *Fagus sylvatica* | PX740800 |
| FS175 | FS_M89 | 2 | *Apiognomonia errabunda* | *Fagus sylvatica* | PX740801 |
| FS177 | FS_M90 | 3 | *Apiognomonia errabunda* | *Fagus sylvatica* | PX740802 |
| FS179 | FS_M91 | 3 | *Gnomoniaceae sp.* | *Fagus sylvatica* | PX740803 |
| FS181 | FS_M92 | 3 | *Didymellaceae sp.* | *Fagus sylvatica* | PX740804 |
| FS183 | FS_M94 | 3 | *Boeremia sp.* | *Fagus sylvatica* | PX740805 |
| FS187 | FS_M100 | 5 | *Dothideomycetes sp.* | *Fagus sylvatica* | PX740806 |
| FS189 | FS_M101 | 4 | *Diaporthe sp.* | *Fagus sylvatica* | PX740807 |
| FS191 | FS_M102 | 3 | *Phacidium sp.* | *Fagus sylvatica* | PX740808 |
| FS193 | FS_M105 | 2 | *Apiognomonia errabunda* | *Fagus sylvatica* | PX740809 |
| FS195 | FS_M106 | 2 | *Clonostachys sp.* | *Fagus sylvatica* | PX740810 |
| FS197 | FS_M109 | 3 | *Sporocadaceae sp.* | *Fagus sylvatica* | PX740811 |
| FS199 | FS_M110 | 2 | *Seimatosporium sp.* | *Fagus sylvatica* | PX740812 |
| FS201 | FS_M113 | 2 | *Clonostachys sp.* | *Fagus sylvatica* | PX740813 |
| FS203 | FS_M114 | 2 | *Fusarium sp.* | *Fagus sylvatica* | PX740814 |
| FS206 | FS_M116 | 5 | *Radulidium subulatum* | *Fagus sylvatica* | PX740815 |
| FS208 | FS_M108 | 1 | *Clonostachys sp.* | *Fagus sylvatica* | PX740816 |
| FS209 | FS_M111 | 3 | *Plagiostoma sp.* | *Fagus sylvatica* | PX740817 |
| FS211 | FS_M112 | 2 | *Sordariomycetes sp.* | *Fagus sylvatica* | PX740818 |
| FS213 | FS_M118 | 3 | *Apiognomonia errabunda* | *Fagus sylvatica* | PX740819 |
| FS214 | FS_M123 | 13 | *Cladosporium sp.* | *Fagus sylvatica* | PX740820 |
| FS216 | FS_M117 | 2 | *Epicoccum sp.* | *Fagus sylvatica* | PX740821 |
| FS221 | FS_M130 | 1 | *Phaeosphaeriaceae sp.* | *Fagus sylvatica* | PX740822 |
| FS225 | FS_M125 | 1 | *Ceratobasidiaceae sp.* | *Fagus sylvatica* | PX740823 |
| FS226 | FS_M122 | 1 | *Cladosporium sp.* | *Fagus sylvatica* | PX740824 |
| FS228 | FS_M136 | 1 | *Didymellaceae sp.* | *Fagus sylvatica* | PX740825 |
| FS234 | FS_M129 | 1 | *Epicoccum sp.* | *Fagus sylvatica* | PX740826 |
| FS242 | FS_M128 | 1 | *Sporocadaceae sp.* | *Fagus sylvatica* | PX740827 |
| FS243 | FS_M140 | 1 | *Diaporthe sp.* | *Fagus sylvatica* | PX740828 |
| FS244 | FS_M93 | 10 | *Cladosporium sp.* | *Fagus sylvatica* | PX740829 |
| FS246 | FS_M98 | 5 | *Penicillium sp.* | *Fagus sylvatica* | PX740830 |
| FS248 | FS_M96 | 4 | *Penicillium sp.* | *Fagus sylvatica* | PX740831 |
| FS252 | FS_M99 | 3 | *Penicillium sp.* | *Fagus sylvatica* | PX740832 |
| FS254 | FS_M95 | 11 | *Penicillium sp.* | *Fagus sylvatica* | PX740833 |
| FS256 | FS_M138 | 1 | *Cladosporium sp.* | *Fagus sylvatica* | PX740834 |
| FS257 | FS_M142 | 1 | *Penicillium sp.* | *Fagus sylvatica* | PX740835 |

## Supplementary Figures

**Supplementary Figure 1.** **Rarefaction analysis of DNA and RNA metabarcoding datasets.** Rarefaction curves are shown for the DNA (A) and RNA (B) datasets, with each curve representing a single sample. Curves are coloured by tree species and exclude singleton taxa.

**Supplementary Figure 2.** **Alpha diversity of fungal communities across methods and tree species.** The Inverse Simpson Index is shown per seed lot comparing (A) different methods and (B) tree species. Bars represent estimated means ± standard errors (SE). The model was run on a rarefied dataset. Different letters above the bars indicate statistically significant differences (p < 0.05).

**Supplementary Figure 3.** **Overlap of fungal OTUs between DNA and RNA metabarcoding across tree species.** Venn diagrams show shared and unique OTUs for Fagus sylvatica (A), Abies alba (B), and Pinus sylvestris (C), based on OTUs from the rarefied dataset.

**Supplementary Figure 4.** **Overlap of fungal OTUs across metabarcoding approaches and tree species.** Venn diagrams show OTU overlap between DNA and RNA metabarcoding (A), among different tree species (B), and between DNA and RNA datasets for Fagus sylvatica (C), Abies alba (D), and Pinus sylvestris (E), highlighting shared and unique OTUs. Diagrams are based on OTUs from the non-rarefied dataset.

**Supplementary Figure 5. Fungal community composition across methods and tree species.** Non-metric multidimensional scaling (NMDS) ordination plots show differences between samples assessed by DNA and RNA metabarcoding (A) and among samples from different tree species (B). Plots are based on Morisita–Horn distances and represent abundance-weighted community patterns (stress = 0.21). Points represent individual samples, coloured by method (A) or tree species (B), with ellipses indicating 95% confidence intervals.

**Supplementary Figure 6. Overlap of fungal genera detected by culturing, DNA, and RNA metabarcoding across tree species.** Venn diagrams show shared and unique genera for Fagus sylvatica (A), Abies alba (B), and Pinus sylvestris (C), based on genera from the non-rarefied dataset.
